# Croup

**Published:** 1883-02

**Authors:** 


### CROUP

**Is inflammation of the wind-pipe, which causes it to be contracted,** making breathing difficult, and sometimes impossible. Croup is the result of cold, though there is generally an hereditary disposition to it.

It comes on with an increased frequency in breathing in the evening ; the next morning the child is better, and at night worse again, and on the third or fourth night, or sooner, it is regular croup. The child is restless, breathes hard, wheezes and has a dry cough. If proper remedies are applied the first or even the second night, but few children will die of croup. Give two teaspoonsful of epsom salts and put the child to bed ; then apply mustard draughts, or "mustard leaves" to its feet. Wring out a flannel cloth in hot water, and wrap it around the neck as warm as it can be borne, protecting the bed with dry cloths. If the breathing is not easier, and the skin not getting moist in 3 or 4 hours, mix half a teaspoonful each of powdered alum and ipecac in half a glass of tepid water and give it. If it does not vomit in ten minutes repeat the dose with a teacup of warm water every five minutes until there is free vomiting. If the bowels are constipated use a "Nelaton Suppository for Children" every 3 hours, until there is a free passage.

If this treatment is applied early, it will seldom fail. If, however, the disease is well established before treatment is commenced, and the above plan of treatment should fail to afford relief in 12 hours, then give ten grains of calomel, mixed with one drachm of saltpetre, called nitrate of potash ; divide into twelve powders and give one every two hours.

*Recapitulation.*—When a child under seven years of age presents symptoms of croup, give two teaspoonsful of epsom salts, put it to bed and apply mustard draughts and cloths wrung out in hot water around the neck. If no decided improvement in three or four hours, give an emetic of half a teaspoonful each of alum and ipecac in half a glass of tepid water, repeating every ten minutes if necessary, until free vomiting is produced.

Every mother should keep on hand, for such an emergency, a bottle of syrup of ipecac, and give two teaspoonsful every ten minutes till free vomiting is induced. This treatment, with good nursing, at the commencement of an attack of croup, will generally be sufficient to effect a cure. During convalescence the little patient should have good nourishment.

### ACID DRINKS.

It is an established principle that cold and hot weather demand clothing and food of a different character, adapted to the varying circumstances. In cold climates and in cold weather the appetite demands, and the comfort of the body justifies, an increased use of carbonaceous food, that the animal heat may be sustained at the vital point of about 78 degrees Fahr. The natural appetite, which is a good guide, demands a change in diet as the spring opens; the disuse of the concentrated foods, the oils, sweets, etc., and the adoption of the succulent products, with those especially containing the sub acids. And, in accordance with this fact, our ever-watchful and kind Provider has given us just the needed medicine foods on the return of warm weather, the berries which are more acid than those appearing later in the season, as all may observe. They are demanded as one means of thinning and purifying the blood, or ridding it of the carbon accumulated during the winter. These acids stimulate the liver to increased activity, removing the waste matter, including that especially from the brain and nerves, which we call "bile," which, in accordance with the same wise and beneficent plan, acts as nature's cathartic, stimulating the bowels, etc., to normal action. It is also true that custom, based on a temporary "craving," demands the use of greens and the like, the prominent feature of which is the acids used—the cooling element. It is unfortunate that in the use of acids we do not select the best, as the natural ones, instead of vinegar, produced by the last or putrefactive stage of the fermentive process, and that in the use of pickles we do not use ripe products, as the beet and the like, instead of the acrid and worthless one-tenth grown cucumber. If we need the acids—as we do—it is far safer to employ them as drinks, as the tartaric acid, the citric and malic, thus avoiding unripe and semi-poisonous fruits. Of the spring medicines I know of none equal to the Acid Phosphate made under the supervision of Professor Horsford, late Rumford Professor at Harvard. It is not merely a medicine, but a food as well, containing, in addition, a pleasant acid—like the "Bread Preparation"—some of the important constituents of the human body. It may be used as a substitute for lemons in drinks, or as a medicine in bilious affections, but is still more efficacious in its action on the brain and nerves, reaching cases of "nervous prostration," so prevalent. It is believed that it may well displace the cathartics, the fashionable "bitters"—often decidedly intoxicating—as well as the "tonics" and stimulants which do so much harm by undue forcing of the system, to be succeeded by a reaction, instead of nourishing the body.

J. H. Hanaford, M. D.

YOUR attention is directed to the advertisement of Caulocorea as prepared under the direction of Warren Lowell, M. D., which is decidedly the most important remedial agent ever discovered for the treatment of all those weaknesses and diseases peculiar to women. The formula of this preparation contains just those drugs which practical experience has verified to be the very best for the conditions for which it is recommended. The very large number of physicians who use Caulocorea testifies to its great value as a nerve tonic and an important therapeutic agent in diseases of the female reproductive organs. It is certainly a sedative and uterine tonic of great power, and is therefore the remedy *par excellence* in painful menstruation or threatened abortion.

AMERICAN TIN MINES.—A correspondent states that tin has been recently discovered in Alabama, under conditions which give every prospect of developing a large and paying industry. For the past two years, Mr. G. W. Gesner, of New York City, has been steadily at work on his lands, about two miles southeast of Ashland, Clay County, Alabama, erecting buildings, opening quarries, and putting up machinery preparatory to mining, washing and smelting the ores of tin found there. The property known as the Broad Arrow mines embraces nearly a square mile, and from its hilly, undulating surface is well adapted to the easiest method of mining. Mr. Gesner has now forty-five stamps working, the first attempt to work tin ore on the spot where found in the United States.

AN artificial wood for various ornamental purposes has lately been introduced in Germany with very satisfactory results. The crude mass from which the articles thus produced are pressed consists chiefly of cellulose mixed with any sort of starch. That is, the ordinary commercial cellulose, which is to be had in any quantity in the form of paper, is softened in water and thoroughly disintegrated, then put in a fine meshed sieve and the water drained off ; it is now mixed with about three parts, by weight, of dry starch, made from wheat, rye, potatoes, Indian corn, etc., as well as two parts of rye or wheat flour, or corn meal, or any other flour that contains gluten, and very intimately mixed.

LONDON is enormous, but the statement that it contains, 5,000,000 people is apt to mislead. What is called the metropolitan area, consisting of land within a radius of fifteen miles of Charing Cross, does contain very nearly that number ; but estimating in this manner from the City Hall, our metropolitan area would probably include 3,000,000. To talk, therefore, of London having 5,000,000 people is delusive.

